# The Significance of Hair for Face Recognition

**DOI:** 10.1371/journal.pone.0034144

**Published:** 2012-03-26

**Authors:** Umar Toseeb, David R. T. Keeble, Eleanor J. Bryant

**Affiliations:** 1 Bradford School of Optometry and Vision Science, University of Bradford, West Yorkshire, United Kingdom; 2 Division of Psychology, University of Bradford, West Yorkshire, United Kingdom; University of British Columbia, Canada

## Abstract

Hair is a feature of the head that frequently changes in different situations. For this reason much research in the area of face perception has employed stimuli without hair. To investigate the effect of the presence of hair we used faces with and without hair in a recognition task. Participants took part in trials in which the state of the hair either remained consistent *(Same)* or switched between learning and test *(Switch)*. It was found that in the *Same* trials performance did not differ for stimuli presented with and without hair. This implies that there is sufficient information in the internal features of the face for optimal performance in this task. It was also found that performance in the *Switch* trials was substantially lower than in the *Same* trials. This drop in accuracy when the stimuli were switched suggests that faces are represented in a holistic manner and that manipulation of the hair causes disruption to this, with implications for the interpretation of some previous studies.

## Introduction

In face processing research a distinction is frequently made between the internal and external features of a face. Internal features are predominantly defined as eyes, mouth, nose, and cheeks (E.g. [1]), and consequently external features are defined as the remaining parts of the face (hair & sometimes chin contour). Sinha and Poggio [2], [3] have presented a widely-cited and striking demonstration of how important external features can be in face perception. In Sinha and Poggio [2], the internal features of the then US-President Clinton were combined with the hair and other external features of his vice-president, Al Gore. The resulting combination appears to casual inspection to be very similar to Gore, implying the dominance of external features. Additionally, in a review by Johnston and Edmonds [4] it has been suggested that the *relative* importance of external and internal features changes as faces become more familiar, with external features being relatively more important in the processing of unfamiliar faces, although this does not imply that external features are unimportant for the recognition of familiar faces.

Longstanding work in face perception indicates the apparent importance of internal features for the perception of *familiar* faces. Ellis, Shepherd and Davies [5] asked participants to identify familiar faces based on either a whole face, only internal features, or only external features. They found that participants were significantly more accurate at recognising such a face with only internal features compared to only external features. Similarly, Young, Hay, McWeeny, Flude, and Ellis [6] broadly concurred with the conclusions drawn by Ellis *et al* [5]. Through the use of a matching task, they found that internal features were matched significantly faster for familiar faces than for unfamiliar faces, hence confirming the heightened preference given to internal features during familiar face matching compared to unfamiliar face matching.

Conversely, the apparent importance of external features for *unfamiliar* faces in measures of accuracy has been demonstrated by two types of experiment: a matching paradigm and a yes/no face recognition paradigm. Bonner, Burton, and Bruce [7] used a matching task to study the time course of the role of internal and external features over 3 days. At baseline when participants were unfamiliar with the faces they performed significantly better at matching faces presented with only external features than they did with only internal features. However, after 3 days performance for the two types of stimuli had become the same. Similarly, Bruce *et al* [8] used a matching task to investigate unfamiliar face recognition and found that participants were significantly more accurate at matching only external features than only internal features. In addition to this, they found that participants were significantly better at matching whole faces compared to faces presented with only external features. Nachson and Schehory [9] again adopted a matching task and found that participants were more accurate at matching unfamiliar whole faces from whole faces compared to external features, which in turn was more accurate than internal features. Hence, the previously mentioned studies [7]–[9] all confirm that at a perceptual level external features are more informative than internal features for unfamiliar faces. More recently, further support for the importance of external features in unfamiliar faces comes from Megreya and Bindemann [10] who again employed a matching task and reported that British participants were more accurate at matching faces from only external features than only internal features. The key difference here was that this importance of external features was only found in British observers and the opposite effect was found in Egyptian observers. Megreya and Bindemann [10] found that Egyptian observers exhibited an internal feature advantage for unfamiliar faces. It could be that face processing strategies vary depending on the kinds of faces people are exposed to. For example, people living in Egypt might acquire an internal feature preference because they regularly come across women wearing a headscarf, whereas the British participants would be less likely to do so.

In these studies, the preference given to external features over internal features in unfamiliar faces emerged from various matching tasks. Ellis *et al* [5] conducted a yes/no recognition experiment where participants were shown a series of faces in the learning phase followed by a test phase consisting of targets and distracters. All participants were presented with whole faces at learning followed by a whole face, only internal features, or only external features at test. It was found that whole faces were recognised significantly better than only internal or only external features. Interestingly, on measures of accuracy no significant difference was found between only internal and only external features. They took this to imply that internal and external features are equally informative in the recognition of unfamiliar faces. A key point to note here is that there is a change in the nature of the stimulus between the learning and test phase in the condition where poorer performance was found, which may affect performance for reasons entirely different from the extent to which internal and external features are actually informative for face recognition. Although the preference towards external features is not explicitly mentioned by these researchers it is evident that in comparison to familiar faces, external features play more of a role in the recognition of unfamiliar faces. There is a substantial body of other research to support the relative importance of external features for the recognition of unfamiliar faces. For example, Lewin and Herlitz [11] used a yes/no recognition paradigm to investigate differences in face recognition abilities, finding that participants performed significantly better when faces were presented with hair compared to when they were presented without hair. Similarly, Wright and Sladden [12] used a yes/no recognition paradigm in which participants were presented with both whole face stimuli and internal feature stimuli during the learning stage. When participants were presented with whole faces at test they found that performance was significantly higher when they had learned whole faces than when they had learned only internal features. Wright and Sladden [12] concluded that hair had a very large effect on the recollection of faces, but again there was a potentially confounding change in stimuli between learning and test phases. The effect of internal and external features was similarly investigated with children [13]. These researchers were able to replicate the findings obtained by Ellis *et al* [5] in adults and in 9 year old children.

Research that uses the yes/no recognition paradigm to investigate the internal/external feature relationship can be conceptualised as being of two types. The first is when the state of each stimulus is kept the same in the learning stage as it was in the test stage (eg, [11]). This will be referred to as the *Same* type. In Lewin and Herlitz [11] participants either viewed a full face or a face with only internal features during the learning phase. During the test phase the participants that viewed a full face at learning also viewed a full face at test and likewise for the internal features. Lewin and Herlitz [11] concluded that participants were significantly more accurate at recognising whole faces compared to faces with only internal features. This implies that external features are important for the recognition of unfamiliar faces and that their absence causes a decrease in performance. Alternatively, the state of the stimuli can be switched between the learning and the test stage, which we denote the *Switch* type. In this variation, the state of the hair between learning and test is changed. For example, Wright and Sladden [12] showed participants faces with either hair or just internal features at learning and during the test phase all faces were shown with hair. Hence, the faces that were shown with no hair at learning and with hair at test were switched. Wright and Sladden [12] found that participants performed significantly better when hair was present in both learning and test compared to when hair was only present at test. Similarly, Ellis *et al* [5], also used the *Switch* method. The matching experiments described above (e.g. [6]) can also be conceptualised as being either *Same*, where the matching images being presented simultaneously are in the same state, or *Switch*, where they are in different states. These studies are almost always performed in the *Switch* mode, with *Same* conditions being included on a fragmentary basis.

It is thus unclear whether the drop in performance in the absence of hair reported in some studies is due to its importance for face perception or purely because of the change between the learning stage and the test stage (or difference in state in a matching task). The results of these studies seem to imply that performance accuracy is due to the presence or absence of hair; however it can be argued that it is due to an alternative factor, namely the change in condition between learning and test stages. It seems that the processing of unfamiliar faces is highly error prone and therefore simple changes in appearance might reasonably be thought to cause a disruption in recognition ability, independent of the extent to which internal and external features are actually needed for optimal recognition performance. An alternative explanation for the decrease in performance when internal features rather than the whole face is used may be that during the learning stage of a yes/no recognition task participants use a variety of configural processes [14] to form a mental representation of a face. Then, when the stimuli are switched between learning and test there is a drop in performance because the test stimuli do not match the mental representation of the face. For this reason it may be that memory for unfamiliar faces is context dependent; therefore a change in context of the inner face causes a disruption to performance. Similar arguments would apply for the matching task results.

Despite the plethora of research into faces and external features, there does not appear to be a study which reports the straightforward conditions of comparing faces with and without hair and the relationship between *Same* and *Switch* conditions, although some of the conditions in the papers previously discussed obviously relate to these issues. Our work used a yes/no recognition experiment in which both the *Same* and the *Switch* conditions were used. Furthermore, the *Switch* conditions were of two types, either switching from/to hair or switching between headscarf and cropped stimuli (neither with hair). We also investigated the effect of race and gender of participants, as these have sometimes been found to be important.

## Materials and Methods

### Ethics

The experiments in this study have been approved by The Biomedical, Natural and Physical Sciences, University of Bradford, Research Ethics Panel. All participants provided written informed consent.

### Participants

Participants were recruited at the University of Bradford using an opportunity sample. A total of 112 participants were used as observers in the experiment. There were 28 South Asian Males (mean age = 21.0 years, SD = 3.3), 27 White Males (mean age = 24.8 years, SD = 6.6), 29 South Asian Females (mean age = 21.9 years, SD = 4.1), and 29 White Females (mean age = 22.1 years, SD = 4.3). The South Asian participants that took part were all British-born.

### Stimuli

**Figure 1:**
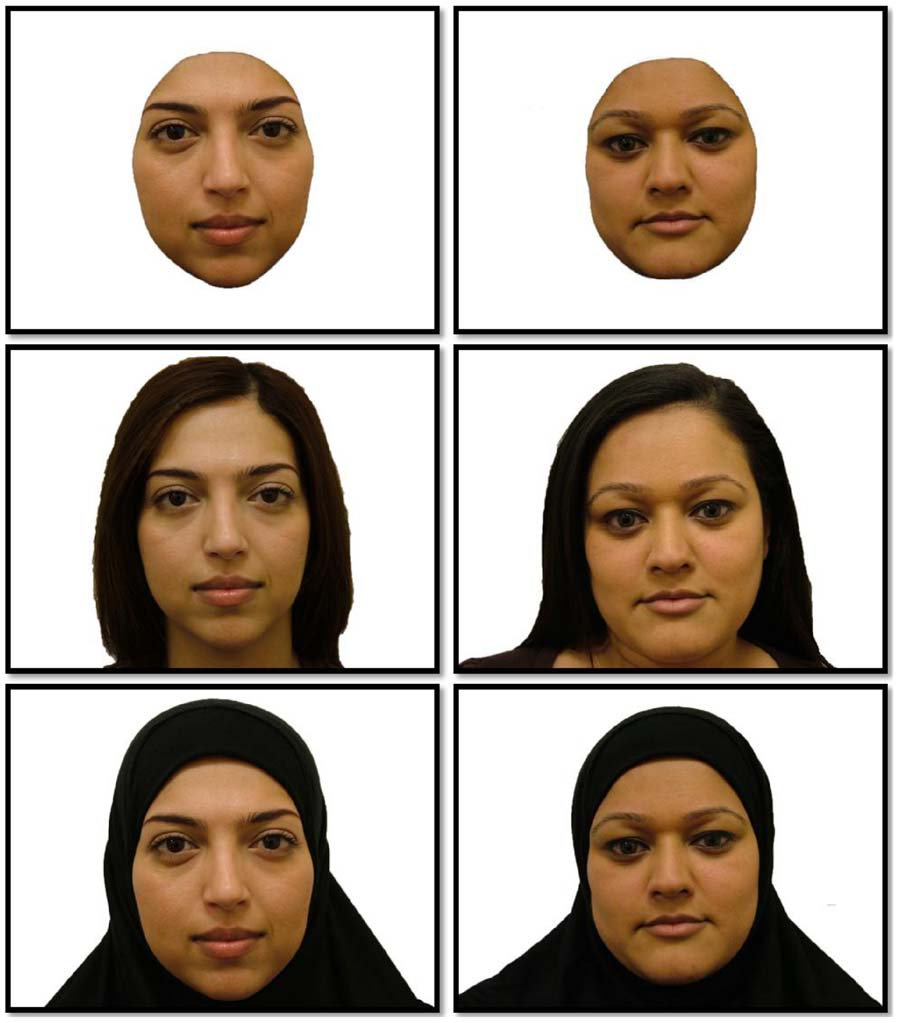
Two faces in different stimulus conditions: Cropped, Hair & Headscarf.

Our research was part of a wider project looking at the effect of the Muslim headscarf on face recognition (Toseeb, Keeble and Bryant, to be submitted). For this reason, the stimuli used in our experiment were only South Asian Females. A total of 24 South Asian females were photographed using a Fujifilm FinePix S7000 digital camera. The age of all participants that were photographed was between 18 and 30 years. Each participant was photographed twice. The first photograph was taken with the participant's hair showing (H). The second photograph was with the participants wearing a Muslim headscarf (HS). All stimuli were then airbrushed using Adobe Photoshop to remove any outstanding features or blemishes. The photograph with the headscarf was then amended to form a cropped face (CR). Participants were photographed with all facial jewellery, makeup, and spectacles removed. When photographing participants it was ensured that there were minimal differences in external factors such as lighting, pose, posture, background, etc. The colour photographs were 1280 pixels×960 pixels with a 32 bit depth. All photographs were then programmed into the E-prime software [15], which was used to run the experiment. See Figure 1 for examples of the stimuli produced using two participants.

### Design

A mixed-subjects design was employed in which the between-subjects variables were Gender (Male or Female), Race (South Asian or White), and Condition (Same* or Switch Hair/Cropped** or Switch Headscarf/Cropped***). The within-subjects variable was State of Stimuli at Test (External Features or No External Features). Participants took part in **only** one of the three conditions. This is shown in Table S1.

*“Same.” refers to the condition in which the stimuli remained the same between the learning and test stage. In this condition participants viewed H and CR faces intermixed in the learning stage. Later in the test stage they were presented with the same stimuli plus distracter faces which had not previously been viewed. The distracter faces were both CR and H.. The participants in this condition took part in H→H and CR→CR trials. In general, we use the nomenclature “X→Y” to indicate that the stimulus was in state X at learning, and state Y at test (because in the subsequent two conditions the stimuli change between the learning and test stage). The total number of participants in this condition was 39 (9 South Asian males, 11 South Asian females, 9 White males, & 10 White females).

**“Switch Hair/Cropped.” refers to the experimental condition in which the stimuli were switched from the learning to the test stage. In this condition participants viewed both H and CR faces intermixed in the learning stage. At test, the external features of previously seen faces were switched. That is, faces that were viewed with hair in the learning stage were now presented as a cropped face and vice versa. The participants in this condition took part in H→CR and CR→H trials. The total number of participants in this group was 36, which was equally divided by gender and race.

*** **“Switch Headscarf/Cropped.”** refers to the experimental condition in which participants viewed both HS and CR faces intermixed in the learning stage. As in the *Switch Hair/Cropped* condition, the state of the external features was switched in the test stage. Therefore, faces that were previously seen with a headscarf were now presented as a cropped face and vice versa. A point of interest here is that none of the faces were presented with hair in either the learning or the test stage. The participants in this condition took part in HS→CR and CR→HS trials. The total number of participants in this condition was 38 (10 South Asian males, 9 South Asian females, 9 White males, & 10 White females).

### Procedure

Participants in all three conditions followed the same procedure. Prior to taking part participants provided informed consent. All instructions were presented on the computer screen to ensure consistency between the different participants and conditions. A yes/no recognition task was used in which participants were presented with a series of pictures in the learning stage and then in the test stage participants were required to decide which faces had been previously seen. All participants were given 8 practice trials with photographs that were not used in the main experiment. The practice trials were used in order to familiarise the participants with the experimental set up. In the main experiment participants were presented with 12 pictures in the learning stage; each for 6000 ms with an inter-stimulus interval of 1000 ms. They were asked to try to remember as many faces as possible as they would be tested later. After the initial presentation of stimuli the participants were given a distracter task (word search) for two minutes. At test participants were presented with 24 pictures (12 previously seen faces and 12 distracter faces) and were required to decide which ones they had seen previously. The distracter faces were always the same individuals regardless of which condition the participants took part in. Each face was presented for 5000 ms or until a response was made. If there was no response detected after 5000 ms a blank white screen appeared until the participant responded. The various experimental timings were determined by pilot experiments in order to reduce ceiling and floor effects. In order to prevent coincidental differences in recognisability of the faces producing a spurious difference in performance between, say, the H-H and CR-CR trials, a form of counterbalancing was employed. For this condition half of the participants would see half of the faces in the H form, with the other half being seen in the CR form. The other half of the participants would see the faces in their complementary forms. In this way each stimulus participant would be seen an equal number of times in each state. This procedure was used for all of the conditions reported here.

## Results

**Figure 2:**
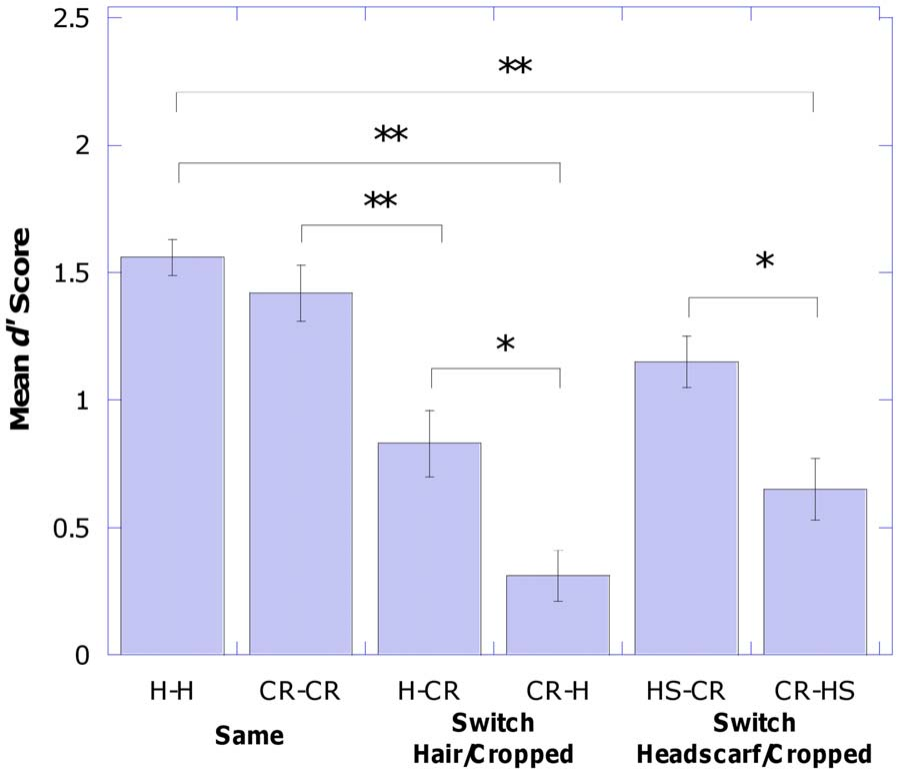
Mean *d′* Score for each of the experimental trials. Data is collapsed across Race and Gender categories. Error Bars represent standard error. H-H refers to those trials in which participants viewed a face with Hair at learning and a face with Hair at test. CR-CR refers to Cropped at learning and Cropped at test. H-CR refers to Hair at learning and Cropped at test. CR-H refers to Cropped at learning and Hair at test. HS-CR refers to Headscarf at learning and Cropped at test. CR-HS refers to Cropped at learning and Headscarf at test. * represents significance at the 0.05 level and ** represents significance at the 0.001 level.

The data produced for each participant was in the form of hits and false alarms for both of the types of trials that the participant took part in. These were converted to the sensitivity score, *d′* [16] and all the analyses were conducted on this measure of sensitivity. The mean and standard deviations are shown in Figure 2.

### Main Effects

A four way mixed ANOVA was conducted in which Gender of Observer (Male or Female)×Race of Observer (South Asian or White)×Condition (*Same*, *Switch Hair/Cropped*, *Switch Headscarf/Cropped*) were entered as between-subject variables and the within-subjects variable was entered as State of Stimuli at Test (External Features and No External Features). Simply for the purpose of this analysis, in the *Switch Headscarf/Cropped* experiment the “HS at test” trials were grouped with External Features and “CR at test” trials were grouped with No External Features. Main effects of State of Stimuli at Test (F (1,101) = 8.491, p = 0.004, partial η^2^ = 0.078) and Condition (F (2,101) = 44.485, p<0.001, partial η^2^ = 0.468) were significant but as they were also involved in further interactions they will not be discussed separately. The main effects of Race and Gender failed to reach the required level of significance, although the main effect of Gender was approaching significance (p = 0.056).

### State of Stimuli at Test×Condition Interaction

A State of Stimuli at Test×Condition interaction (F (2,101) = 4.965, p = 0.009, partial η^2^ = 0.090) was observed. To investigate this interaction further, three paired samples t-tests were conducted which looked at the difference between the two types of stimuli within each condition. The first of these three t-tests found that there was no significant difference between Hair and Cropped stimuli in the *Same* condition (t (38) = 1.022, p = 0.313). Hence, there was similarity in the accuracy of performance between the Hair and Cropped stimuli when the state of the stimuli remained the same between the learning stage and the test stage. The second t-test looked at the *Switch Hair/Cropped* condition and found that participants performed better when they viewed no hair (i.e. CR) at test (H→CR) than when they viewed hair at test (CR→H) (t (36) = 2.624, p = 0.013). Thus, the addition of hair on to a previously viewed Cropped face resulted in poorer performance compared to the removal of hair from a face previously viewed with hair. Finally, The t-test looking at the *Switch Headscarf/Cropped* condition found that participants performed significantly better when they viewed no hair (i.e. CR) at test (HS→CR) than when they viewed a headscarf at test (CR→HS) (t (38) = 3.777, p = 0.001). Therefore, the addition of a headscarf on to a previously viewed cropped face led to a worsening of performance compared to the removal of a headscarf from a face previously viewed with a headscarf.

An alternative interpretation of this interaction examined the difference between each of the conditions separately for the two types of trial in each condition. Hence, 2 One Way ANOVA's were conducted. The first, which examined NoHair at Test stimuli was significant (F (2,110) = 12.710, p<0.001), so Bonferonni post-hoc comparisons were conducted. The comparisons showed that performance for those participants in the *Same* condition (CR→CR) did not differ to those in the *Switch Headscarf/Cropped* condition (HS→CR), p>0.05, however it was significantly higher than those in the *Switch Hair/Cropped* condition (H→CR), p<0.001. In addition to this, there was no significant difference between participants in the Switch Hair/Cropped condition (H→CR) and Switch Headscarf/Cropped condition (HS→CR), p>0.05.This finding was also replicated in the second One Way ANOVA which looked at the Hair at Test stimuli (F (2,110) = 42.381, p<0.001). Bonferonni post hoc comparisons showed that participants in the *Same* trials (H→H) performed significantly higher than those in the both *Switch Hair/Cropped* and *Switch Headscarf/Cropped* trials (CR→H (p<0.001) & CR→HS (p<0.001)). However, Switch Hair/Cropped and Switch Headscarf/Cropped did not differ from each other (p>0.05).

### Race×Condition Interaction

**Figure 3:**
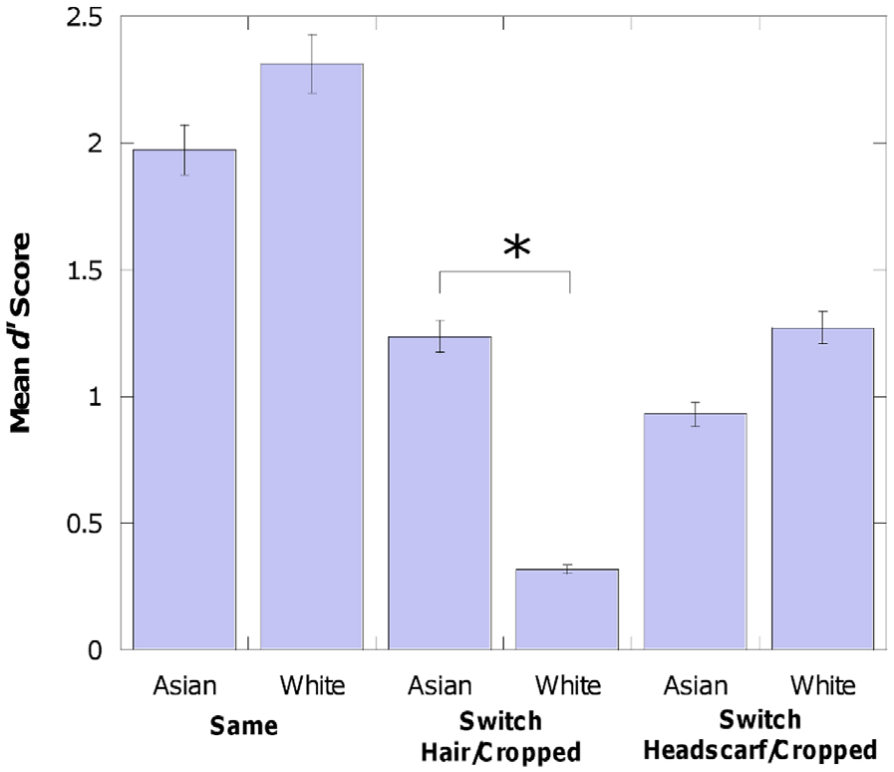
Mean *d′* Score for each of the conditions split by Race. Data is collapsed across Gender. Error Bars represent standard error. * represents significance at the 0.001 level.

A Race×Condition interaction also reached significance (F (2, 101) = 11.221, p<0.001, partial η^2^ = 0.182). To investigate this further, three independent t-tests were conducted which looked at the difference between South Asian and White participants in each of the 3 conditions. It was found that in the *Switch Hair/Cropped* condition South Asian participants performed significantly better (difference in *d′* of 0.92) than the White participants (t (34) = 4.330, p<0.001). No such differences were found for the *Same* (t (37) = 1.608, p = 0.116) or the *Switch Headscarf/Cropped* (t (36) = 1.694, p = 0.099) conditions. The breakdown of this is shown in Figure 3 and Table 1.

**Table 1:** Mean *d′* and *(SE)* split by each of the ethno-gender categories.

	Same		SwitchHair		SwitchNoHair	
	H-H	CR-CR	H-CR	CR-H	HS-CR	CR-HS
Asian Male	2.33 *(.85)*	1.72 *(1.25)*	1.29 *(.90)*	.67 *(1.02)*	1.00 *(.79)*	.50 *(.92)*
White Male	2.26 *(.65)*	2.09 *(.79)*	.52 *(.87)*	.05 *(.69)*	1.45 *(.61)*	.79 *(.95)*
Asian Female	1.87 *(.77)*	1.98 *(1.09)*	1.72 *(.87)*	1.27 *(1.04)*	1.39 *(1.09)*	.83 *(1.33)*
White Female	2.47 *(.74)*	2.44 *(.75)*	.81 *(.92)*	−.11 *(.99)*	1.74 *(.66)*	.11 *(.79)*

## Discussion

### Same Condition

It transpired that the presence of hair *per se* did not aid recognition. When the stimuli were kept the same between learning and test there was no significant difference in performance between faces presented with and without hair. Our findings were somewhat different to those of Lewin and Herlitz [11] who found that participants performed better when stimuli were presented with hair even when the stimuli were kept constant between learning and test. One of the key differences between our experiment and that of Lewin and Herlitz [11] was that we employed a within-subjects design whereas Lewin and Herlitz [11] used a between-subjects design. We propose that when faces are learnt in a within-subjects design where each subject sees both whole faces and cropped faces intermixed, internal features play a dominant role because they are the only set of features which are present in all the different faces. Thus, attention is generally more focused on the internal features. In a between-subjects design this is not the case because the participants in the Hair condition would have more information to potentially use and hence would use the hair as a cue whilst the participants in the No External Features condition could not. We repeated the same experiment using a between-subjects design (Toseeb, Keeble, Wickham, & Bryant, in preparation) and found a small but statistically significant difference in the same direction as Lewin and Herlitz [11]. This suggests that encoding mechanisms may differ somewhat depending on the type of design that is used. This study appears to be the only pre-existing study which allows any direct comparison to be made between the “H-H’ and “CR-CR” tasks, and employed rather different stimulus conditions to ours.

To understand our findings further it is necessary to explore what other researchers have used as “only internal features”. Leder and Carbon [17] presented participants with both individual features (eyes, nose, or mouth) and whole faces (with hair) during the learning stage of a recognition task. Then during the test stage participants were again presented with both individual features and with whole faces. Participants performed better in the whole face condition than they did in the part face condition. These findings support the whole-part superiority effect proposed by Tanaka & Farah [18], who claim that parts of the face are recognised better in the context of the whole face rather than on their own. However, we did not find a whole-part superiority effect presumably because our “cropped” stimuli included much more information than just the eyes, nose, or mouth used by the aforementioned research [17], [18]. There are three different types of potential stimuli that can be used: whole faces (including hair), cropped faces, and individual internal features. The comparison made by the other studies [17], [18] is of whole faces versus individual internal features whereas our research compares whole faces with cropped faces. Therefore, our research suggests that although performance may not be optimal when internal features are presented individually, when they are presented in the context of the face along with the other internal features, then performance is the same as when they are presented in a face with hair.

Our results are compatible with fMRI data from Betts and Wilson [19] who found that there was no difference in activation of the fusiform face area (FFA) for whole faces compared to only internal features (face minus the hair). The fusiform face area (FFA) is thought to be an area of the brain which has some responsibility for the processing of faces [20]. Betts and Wilson's [19] result might suggest that the FFA may process a face without hair in the same manner as a face with hair.

### Switch Hair/Cropped Condition

It was also found that when the stimuli were switched to or from hair between learning and test, performance was lower compared to when they remained the same. That is, when the faces were learnt with hair and then tested without hair, performance was lower than when they were tested with hair. These findings are in fact compatible with the research discussed previously (e.g. [5], [12]) however, our explanation of this effect is somewhat different. Whereas those researchers attribute the drop in performance when hair is removed (H-CR worse than H-H and CR-H worse than H-H, respectively) to the loss of the information in the external features, we believe that the fact that our additional task condition CR-CR is performed at the same level as H-H implicates the change between the state of the stimulus, rather than any putative additional information provided by the hair. In other words, the switch disrupts holistic processing. This result suggests that a number of other earlier results discussed above which had been taken to support the importance of external features are actually due to a change in stimulus between learning and test.

It would be interesting to replicate our study using alternative methodologies, including matching paradigms [6] and a modified yes/no paradigm where the learning and test images are slightly different, thereby obviating the possibility of image matching strategies being used, although as Sporer [21] points out, this latter method is only rarely employed.

We again refer to results from fMRI face perception studies in an attempt to show that our findings are consonant with the workings of underlying physiological mechanisms. Andrews, Davies-Thompson, Kingstone, and Young [22] used an adaptation paradigm in which participants took part using four different types of image conditions. They saw a sequence of pictures of faces with (1) same internal features/same external features, (2) same internal features/different external features, (3) different internal features/same external features, and (4) different internal features/different external features. It was found that the same internal/same external condition elicited a lower activation in the FFA than in the other three conditions. Therefore, along with Axelrod and Yovel [23] and Betts and Wilson [19], Andrews *et al* [22] found that a change in either internal or external features causes a release in adaptation in the FFA. This in turn may suggest that internal and external features are not independently represented in the FFA but rather, they are processed interactively and the face is represented holistically. This would explain why when external features of successive images were changed, there was a release from adaptation. During our recognition task, the change of external features between the learning and the test stage may have caused a disruption in performance due to faces being processed holistically. Therefore, if the parts of the face that are present at the time of learning are consistent with those parts presented at test, then hair is not required for optimal performance. However, in the case where the parts of the face at learning are inconsistent with the parts at test (e.g. CR→H) then hair can have a detrimental effect on performance. This explanation is also compatible with the encoding specificity principle [24] which states that cues at test will be most effective if they match those that were present at the time of learning. In this way, in the research by Leder and Carbon [17] participants took part in both *Same* and *Switch* conditions. Leder and Carbon [17] found that performance was better with full faces when full faces were learned and performance was better with part faces (eyes, nose, or mouth) when part faces were learned. Our results support Leder and Carbon [17] in showing that if the image at test is not compatible with the representation in memory then performance suffers regardless of whether it is a whole face or individual features.

Some other results can be predicted purely on the basis that a change in stimulus between learning and test impairs performance. For example, Patterson and Baddeley [25] conducted recognition experiments in which the state of hair and wigs in males was kept the same or switched between learning and test. They found that participants performed significantly worse when a change was made either hair, wig, or both from learning to test, compared to when there was no change. It is not just manipulation of hair that may cause a disruption in performance. Buttle and East [26] found that during a recognition task when participants learnt a normal upright face (with hair) and then tested with either the same face, same face with half-covered in Maori tattoos, same face completely covered in Maori tattoos, or inverted face, the performance was significantly disrupted with the addition of tattoos compared to the normal face. Performance levels fell to the equivalent of the inverted face in the full tattoo condition, implying that holistic processing is disrupted to a similar extent by both manipulations [27], [28]. Similarly, Ueda and Koyama [29] used a matching task to explore the effects of facial makeup. They found that heavy facial makeup disrupted performance compared to the same face without makeup. In neither the work of Buttle and East [26], nor of Ueda and Koyama [29] is it possible to disentangle the effects of change of state from the effects of the state of the face as such, because the full set of comparison conditions was not performed. Therefore, although the authors of these two papers attribute the fall in performance solely to the presence of the additional feature, it may simply have been due to the change in feature.

Furthermore, our results showed that in the trials where the state of stimuli was switched (with hair) the addition of hair from learning to test caused more disruption than the removal of hair to produce a cropped stimulus. In the *Switch Headscarf/Cropped* condition it was found that, again, the addition of a headscarf from learning to test caused more of a disruption than its removal. Leder and Carbon [17] provide one explanation for why the addition of features (hair or headscarf) would cause more disruption than their removal. They propose the concept of holistic interference, in that the context of the whole face at test during *Switch* conditions interfered with the parts of the face which are represented in memory. Furthermore, Murray and Jones [30] suggest that irrelevant information is automatically processed up to a semantic level and hence causes interference. Therefore, the change between learning and test causes disruption because the holistic representation of the face is affected, and when the change involves the addition of irrelevant information then more disruption occurs because it is difficult to ignore irrelevant information.

### Switch Headscarf/Cropped Condition

Broadly speaking, the results for this condition are similar to that of the *Switch Hair/Cropped* condition, in that performance is worse than for the *Same* condition, presumably because again there is a change in state between learning and test. Although, the difference between HS→CR and CR→CR did not quite achieve significance, it seems most likely to us that there is in fact a real difference between the two conditions, but that this difference is smaller than between the *Same* and *Switch Hair/Cropped* conditions. A potential explanation for this might be that the headscarf images are somewhat less complex than the hair images, and therefore may not have provided participants with sufficiently rich information during learning, and so may not have been processed to the same extent. So when a face with a headscarf is learnt, the headscarf is processed less compared to hair due to the reduction in information that it gives. Hence, the holistic interference that actually occurs is less severe because the headscarf was processed very shallowly.

Although our study was not primarily aimed at investigating the Own Race Bias (ORB), some of our results bear on this issue. Meissner and Brigham [31] in their meta-analysis of the ORB report that the vast majority (88%) of samples used were either White or Black, with only a few studies employing other races. According to the Office for National Statistics [32] people from a South Asian origin constitute 4.4% of the British population. However, it appears that only Walker and Hewstone [33]–[35] have investigated the ORB using a South Asian population in the United Kingdom. They found that the ORB was present in White observers but not those from a South Asian background. Our work has indirectly explored the same issue using a sample from the University of Bradford where the population of South Asian students is approximately 32.1% [36]. People of South Asian origin constitute 26.1% of the population of the city of Bradford [32]. On the investigation of the ORB an effect was only found in the “*Switch Hair/Cropped*” condition. In this condition South Asian participants performed significantly better than the White participants. This result was not seen in either of the other two conditions. It may be that hair was used as an indicator of race, however this is only speculative. As shown from the previous findings, in the *Same* condition hair was not a contributing factor in performance and performance was relatively good. However, when the task became more difficult in the *Switch Hair/Cropped* experiment, hair was available to use as an indicator of race and therefore it can be hypothesised that White participants perhaps more readily categorised the faces as the out-group causing a difference in performance. This difference was not replicated in the *Switch Headscarf/Cropped* experiment presumably because in line with Leder and Carbon [17] although the irrelevant external features are automatically processed up to a semantic level, they are not as deeply processed as hair therefore the out-group categorisation does not occur. Additionally, our results are compatible with the Own Gender Bias [12] in which subjects are better at recognising other people of the same gender compared to people of the opposite gender: we found that females were slightly (but not quite significantly) better than males at recognising female faces.

### Conclusions

We have shown that the presence or absence of hair does not generally affect the ability to recognize unfamiliar faces when there is consistency between the learning and test phases of the experiment. However, due to the holistic nature of face processing, manipulation of external features can sometimes disrupt face recognition, providing an alternative explanation for a number of previous results.

## Supporting Information

Table S1 **Different types of trials in each of the experimental conditions. Participants took part in only one of the three conditions.**
(DOC)
