# Supplementary material for: The Significance of Hair for Face Recognition
Source: PLoS One. 2012 Mar 26;7(3):e34144. doi: 10.1371/journal.pone.0034144 (PMC3312903; doi:10.1371/journal.pone.0034144)
Supplement: Table S1 — Different types of trials in each of the experimental conditions. Participants took part in only one of the three conditions. (DOC) [file pone.0034144.s001.doc]

| **Condition** | **Learning** | **Test** |
| --- | --- | --- |
| Same | 6 Faces with Hair  (H)  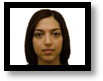 | 12 Faces with Hair  (H)  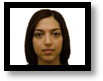 |
|  | 6 Faces with Hair Cropped (CR)  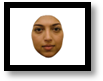 | 12 Faces with Hair Cropped (CR)  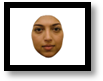 |
| Switch Hair/Cropped | 6 Faces with Hair  (H)  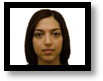 | 12 Faces with Hair Cropped (CR)  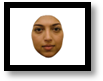 |
|  | 6 Faces with Hair Cropped (CR)  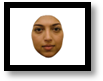 | 12 Faces with Hair  (H)  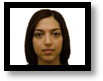 |
| Switch Headscarf/Cropped | 6 Faces with a headscarf (HS)  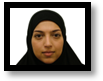 | 12 Faces with Hair Cropped (CR)  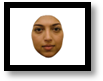 |
|  | 6 Faces with Hair Cropped (CR)  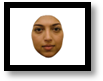 | 12 Faces with a headscarf (HS)  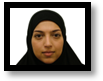 |
